# Effective number of white shark (*Carcharodon carcharias,* Linnaeus) breeders is stable over four successive years in the population adjacent to eastern Australia and New Zealand

**DOI:** 10.1002/ece3.7007

**Published:** 2020-12-09

**Authors:** Danielle Davenport, Paul Butcher, Sara Andreotti, Conrad Matthee, Andrew Jones, Jennifer Ovenden

**Affiliations:** ^1^ Molecular Fisheries Laboratory and Schools of Biomedical Sciences University of Queensland St. Lucia QLD Australia; ^2^ New South Wales Department of Primary Industries Coffs Harbour NSW Australia; ^3^ Evolutionary Genomics Group Department of Botany and Zoology Stellenbosch University Stellenbosch South Africa

**Keywords:** conservation, effective number of breeders, population genetics, effective population size, monitoring

## Abstract

Population size is a central parameter for conservation; however, monitoring abundance is often problematic for threatened marine species. Despite substantial investment in research, many marine species remain data‐poor presenting barriers to the evaluation of conservation management outcomes and the modeling of future solutions. Such is the case for the white shark (*Carcharodon carcharias*), a highly mobile apex predator for whom recent and substantial population declines have been recorded in many globally distributed populations. Here, we estimate the effective number of breeders that successfully contribute offspring in one reproductive cycle (*Nb*) to provide a snapshot of recent reproductive effort in an east Australian–New Zealand population of white shark. *Nb* was estimated over four consecutive age cohorts (2010, 2011, 2012, and 2013) using two genetic estimators (linkage disequilibrium; LD and sibship assignment; SA) based on genetic data derived from two types of genetic markers (single nucleotide polymorphisms; SNPs and microsatellite loci). While estimates of *Nb* using different marker types produced comparable estimates, microsatellite loci were the least precise. The LD and SA estimates of *Nb* within cohorts using SNPs were comparable; for example, the 2013 age cohort *Nb*(*SA*) was 289 (95% CI 200–461) and *Nb*(*LD*) was 208.5 (95% CI 116.4–712.7). We show that over the time period studied, *Nb* was stable and ranged between 206.1 (*SD* ± 45.9) and 252.0 (*SD* ± 46.7) per year using a combined estimate of *Nb*(*LD+SA*) from SNP loci. In addition, a simulation approach showed that in this population the effective population size (*Ne*) per generation can be expected to be larger than *Nb* per reproductive cycle. This study demonstrates how breeding population size can be monitored over time to provide insight into the effectiveness of recovery and conservation measures for the white shark, where the methods described here may be applicable to other data‐poor species of conservation concern.

## Graphical abstract

Population size is a central parameter for conservation; however, monitoring abundance is often problematic for threatened marine species. In this study, the effective number of breeders was estimated over time for a population of white shark. We show how the breeding population size can be monitored over time and provide insights into the effectiveness of recovery and conservation measures for this species. Photo credit: Dr Paul Butcher.

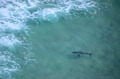

## 1 INTRODUCTION

Assessing the size of natural populations is a key objective of monitoring programs which are vital for understanding the conservation status of species, the regulating effects of biotic and abiotic factors, and for the assessment of management efforts (Lindenmayer et al., 2020). However, for many marine populations there is a lack of consistent monitoring programs at appropriate spatial and temporal scales for conservation and policy needs (Papa et al., 2020). This presents a significant problem for chondrichthyans (sharks, skates, rays and chimaeras), where more than half of known species are characterized by insufficient data and one‐quarter are estimated to be at risk of extinction (Dulvy et al., 2014). Within the elasmobranchs (sharks, skates, and rays), each contributes significantly to connect ecosystems and regulate marine food webs (Heupel et al., 2014). However, habitat loss and continued pressures on mortality though bycatch and targeted fishing have resulted in many populations of elasmobranch being depleted at a rate that exceeds their natural recovery potential (Worm et al., 2013). Given the significant challenges facing elasmobranchs and the importance of their role in regulating marine ecosystems, improvements for monitoring changes in natural populations are critical.

Monitoring threatened elasmobranch species is particularly challenging for many reasons. In the case of the white shark, *Carcharodon carcharias* (Linnaeus, 1758), where monitoring is a both a social and conservation priority, efforts to evaluate long‐term population trends have been hampered by issues including detectability [misidentification in photo‐ID surveys (Burgess et al., 2014), lack of resightings in mark‐recapture studies (Gore et al., 2016), effects of environment on heterogeneity of behavior (Jacoby et al., 2012)] and a lack of catch statistics (Roff et al., 2018). The need for alternate methods to index shark populations has therefore led to the increasing use of molecular markers to evaluate change and inform management (Blower et al., 2012; Bruce et al., 2018; Hillary et al., 2018). In this study, we focus on the concept of genetic effective population size (herein effective population size—*Ne*), which can be used to evaluate change in abundance from allele frequencies (Schwartz et al., 2007). When populations are small, genetic models predict that the evolutionary force of genetic drift (stochastic changes in allele frequencies) will predominate over other evolutionary forces such as natural selection, to reduce genetic diversity, population viability, and evolutionary potential (Frankham, 1996; Franklin, 1980). The extent to which a population is vulnerable to such effects is inversely related to the magnitude of *Ne*, where the effects of drift will occur more slowly in populations with larger effective sizes than those with smaller effective sizes (Wang, 2005). When a genetic sample contains only individuals from a single age cohort (a group of individuals having the same age‐class), then the estimate of *Ne* corresponds to the effective number of breeders (*Nb*) which contributed offspring to that cohort (Wang et al., 2016; Waples et al., 2013). For long‐lived, iteroparous species, estimates of *Nb* are generally considered more useful for monitoring as they apply to a single breeding season and represent an accessible parameter for monitoring population trends at ecological timescales most relevant to conservation and management needs (Ovenden et al., 2016; Schwartz et al., 2007; Waples & Do, 2008). Past research has confirmed the power and usefulness of *Nb* as a tool to monitor population trends (Antao et al., 2011; Luikart et al., 2020; Nunziata & Weisrock, 2018). For instance, quantifying changes in *Nb* over time provides high power to detect declines in *Nb* *(Luikart et al.,* 2020 *)* and has helped to identify factors relevant to shaping populations (i.e., management interventions, demographic parameters) with successful outcomes reported for populations of commercially important bony fishes. Examples include salmon (Bacles et al., 2018; Perrier et al., 2016), trout (Ruzzante et al., 2019; Whiteley et al., 2013; Wood et al., 2014), snapper (Jones et al., 2019), and tuna (Waples et al., 2018). In these examples, both *Nb* and *Ne* were used to investigate demographic (i.e., variance in reproductive success under commercial harvest conditions) and environmental (i.e., stream productivity, competition, habitat quality, year‐of‐the‐young development) effects on long‐term population viability, with significant implications for management and conservation.

In this study, we trialed a sampling and genotyping protocol aimed at estimating *Nb* over time (four breeding seasons; 2010–2013) in a population of *C. carcharias* of conservation concern. We focus on the east Australia–New Zealand population (EAP) of *C. carcharias* which, due to patterns of coastal residency and site fidelity (Bruce et al., 2019; Spaet et al., 2020), is genetically distinct from other identified populations in the North‐Pacific, South‐West Australia, Atlantic, South Africa, and Mediterranean (Andreotti et al., 2016; Blower et al., 2012; Gubili et al., 2010; O’Leary et al., 2015; Tanaka et al., 2011). The EAP has experienced a large (>90%) decline during the 20th century due to targeted fishing and mortalities associated with bather protection programs (Reid et al., 2011; Roff et al., 2018); however, recovery is now anticipated due to protection through international conventions and jurisdictional legislation [i.e., International Plan of Action for the Conservation and Management of Sharks (FAO, 2000) and the Environment Protection and Biodiversity Conservation (EPBC) Act of 1999 (EPBC, 1999)]. Previous efforts to detect population recovery using historical catch data (Roff et al., 2018) and genetic close‐kin mark–recapture (Bruce et al., 2018; Hillary et al., 2018) found no significant evidence of population growth or recovery in the EAP. Updated bather protection programs along parts of east coast Australia (i.e., 'Shark Management Alert in Real Time' (SMART) drumlines, see Tate et al., 2019), aimed at minimizing unfavorable interactions with marine environment users, offer an opportunity for nonlethal tissue sampling and to determine the usefulness of this genetic monitoring method in the EAP. Our specific objectives were to: (a) use two genetic methodologies to estimate *Nb* over time in the EAP [sibship assignment (SA) (Wang, 2009) and linkage disequilibrium (LD) (Hill, 1974, p. 197; Waples, 2006)]; (b) validate these results using two types of nuclear genetic markers (single nucleotide polymorphisms and microsatellites); (c) investigate Nb/N ratios using published estimates of the adult population size (*Na*); and (d) develop expectations for generational *Ne* in the EAP using life‐history information and simulations. Our results for the EAP of *C. carcahrias* suggest that Nb has not changed significantly year‐to‐year and provides insight into the effectiveness of recovery and conservation measures following historical declines.

## 2 METHODS

### 2.1 Samples

**Figure 1:**
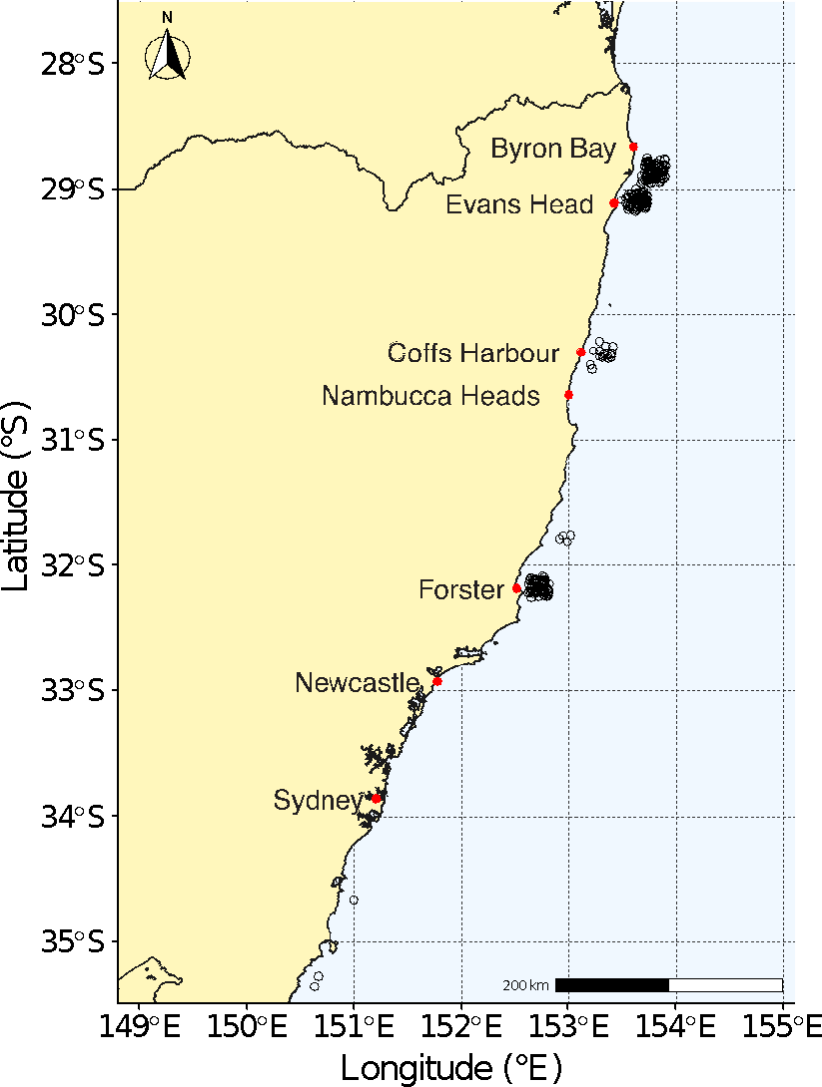
Map of sampling locations, where 247 EAP samples (open circles) were collected along the NSW coast and used to determine *Nb*. Red dots indicate location of named places

To obtain genetic data to estimate *Nb* in the east coast Australia–New Zealand population (EAP) of *C. carcharias,* tissue samples (*n* = 247) were nonlethally collected during 2015 to 2018 from juvenile and subadult *C. carcharias* between Buckley Beach, Narrawallee (−35.29873, 150.48331), and Seven Mile Beach, Lennox Head (−28.76130, 153.62020) (Figure 1). Individuals were captured, restrained, tagged, and released as part of the New South Wales (Australia) Shark Management Strategy. Fin clips were collected for genetic purposes and fork length (FL) and total length (TL) measurements were taken from each individual. Since migration between populations can bias genetic estimates of both *Ne* and *Nb* (Macbeth et al., 2011), the population of origin for each individual was resolved through the inclusion of tissue samples of white sharks collected from other locations (Western Australia *n* = 3; South Australia *n* = 9; South Africa *n* = 20; total *n* including EAP samples = 279, see Table S1). All samples were used in the SNP discovery and genotyping pipeline.

### 2.2 Cohort assignment

To group individuals into age cohorts, a year‐of‐birth was assigned to each sample using the year the individual was sampled minus the age of the individual in that given year. To estimate the age of individuals, we used the von Bertalanffy growth function (VBGF) (Supplementary Material S2) to transform the relationship of TL to relationships at age using growth parameters specific to the EAP (O’Connor, 2011). We considered fork length (FL, defined here as the measurement from the tip of the rostrum to the fork in the tail over the body) the more accurate measurement at the time of sampling. Conversion of FL to TL was achieved by linear regression based on measurements of study samples using the *lm* function in R (O’Connor, 2011). Assumptions of linearity, normality, and heteroscedasticity were checked by means of residual and quantile plots and the following conversion was used to transform measurements:

(1) $$TL\left(cm\right)=6.80+FL\left(cm\right)*1.07$$

### 2.3 SNP and microsatellite loci datasets

DNA was extracted from all samples (*n* = 279) using a standard salt precipitation procedure. For SNP data, the samples were genotyped by DArT P/L laboratory using DArTseq^TM^ technology (Kilian et al., 2012). Sequencing steps followed Kilian et al., (2012) and were completed using an Illumina HiSeq 2500. Resulting sequences were processed using the proprietary DArT analytical software, DArTsoft14. DArTsoft14 uses technical sample replicates to optimize its algorithm parameters and ensure scoring consistency (see Georges et al., 2018). Postprocessing of SNPs was completed in R (R Core Team, 2018) using the R‐Package *radiator 0.0.5* (Gosselin, 2017) and custom R‐scripts following current best practice (O’Leary et al., 2018; Shafer et al., 2017). A two‐stage postprocessing approach was applied to the SNP dataset to identify and remove 1) migrants and 2) outlier loci (candidate loci under selection, so as to retain only nonselective neutral loci). SNP data representing all samples (East Australia, South Africa, Western Australia, South Australia) were filtered following the steps outlined in Table S3.1 (Supplementray Materials S3), and subsequently used for sample population assignment and initial outlier loci discovery. Strongly divergent individuals create strong mixture LD which downwardly bias estimates of *Ne*(*LD*) (Waples & England, 2011) and may contribute to upward bias in estimates using the SA method (Ackerman et al., 2017). To identify divergent individuals, we performed a discriminant analysis of principal components (DAPC) (Jombart et al., 2010) implemented in the R‐package *adegenet* (Jombart et al., 2010). The optimal number of discriminant functions to retain was calculated using the function *xvalDAPC* using 80% of the data in the training set, and the number of PCs retained in the final DAPC was associated with the lowest mean squared error. As indicated in Figure S3.1, two samples collected from east Australia appeared distinct from other EAP samples (subsequently confirmed using tracking data from acoustic tagging, Spaet et al., 2020). These samples were removed from subsequent analysis. We also performed tests for putitative loci under selection which deviate from the assumptions necessary for estimating *Ne* (Waples & England, 2011). We used *pcadapt* (Luu et al., 2017) which identifies outlier loci in a multidimensional space (we used *k* = 3 principal components). We removed loci when the q‐value (test statistic) was smaller than the false discovery rate (α=0.05). In the second stage of SNP postprocessing, we used a dataset (herein Dataset‐2) containing all SNP loci except those identified as selective outliers and including only samples representing genotypes of EAP origin only. We then filtered Dataset‐2 using reproducibility greater than 98%, a minor‐allele‐count greater than three, coverage (minimum 5, maximum 25), retained only one SNP per locus and removed individuals missing greater than 20% of SNP loci. Loci were further removed where Hardy–Weinberg disequilibrium mid‐p was less than 0.01α<0. and if *F_IS_* was greater than or equal to +0.5 or less than or equal to −0.5 (see Table S3.2). Dataset‐2 was then used to make estimates of *Nb*.

Extracted DNA from 192 EAP samples was further genotyped in another laboratory (Stellenbosch University) with nineteen species‐specific microsatellite loci to provide alternate estimates of *Nb*. Fourteen of the loci were derived from previous studies: Ccar1, Ccar13, Ccar6.27x, Ccar9, Iox10, Cca1419, Cca83, Cca1536, Cca1273, Cca711, Cca1072, Cca1466, Cca1276, and Cca1226 (Gubili et al., 2010; O’Leary et al., 2015; Pardini et al., 2001). Five loci (CcSA1, CcSA2, CcSA3, CcSA4, and CcSA5) were developed using the methods described in Andreotti et al. (2016). Wet laboratory genotyping was performed as described by Andreotti et al. (2016), and genotype scoring was performed in *Geneious* v.5.6.5 (©2005–2012 Biomatters Ltd). Assessment of amplification errors, such as large allele drop‐out, stuttering, and null alleles, was conducted in *Microchecker* v.2.2.3 (Van Oosterhout et al., 2004). The program SHAZA (Macbeth et al., 2011) was used to detect duplicates in the dataset. Descriptive statistics, including observed heterozygosity (H_o_) and expected heterozygosity (H_e_), were calculated using the R‐package *hierfstat* (Goudet et al.,^2018^). Hardy–Weinberg equilibrium (HWE) was evaluated using an exact test based on 10,000 Monte Carlo permutations of alleles and implemented in *Genepop* (Rousset, 2008).

### 2.4 Estimation of Nb

Two methods were used to estimate *Nb* from data derived from either SNP or microsatellite loci: (a) the linkage disequilibrium method (LD) (Hill, 1974; Waples, 2006) and (b) the sibship assignment method (SA) (Wang, 2009). Estimates are referred to as *Nb*(*LD*) and *Nb*(*SA*). Broadly, the LD method determines the size of the parental generation using a measure of the genetic association (or LD) in a given age cohort. In finite populations, random genetic drift leads to associations of alleles at different loci. The LD method uses the extent of nonrandom association between alleles at different loci to estimate genetic *Ne* and reflects the inbreeding *Ne* when loci are unlinked (Hill, 1981; Waples & Do, 2010). The formulation of the LD method uses the observed average disequilibrium between pairs of independent (i.e., nonlinked), neutral loci in a sample of individuals taken from a single, isolated, randomly mating population. Estimates of *Nb*(*LD*) are based on the theoretical relationship between ${\hat{r}}^{2}$and *Ne* as described in Hill (1981);

$$\hat{N_{e}}=\frac{1}{3({\hat{r}}^{2}‐\frac{1}{S})}$$

Equation 2a from (Waples & Do, 2010)

where r^2^ is the mean squared correlation of allele frequencies at different gene loci adjusted for sampling error (i.e., the observed average disequilibrium) and *S* is the number of individuals sampled. We implemented this method using the program *NeEstimator* v2.1 (Do et al., 2014). In contrast, the SA method uses the direct relationship between genetic relatedness and inbreeding *Ne*, such that any two individuals sampled randomly from a population with a small *Ne* will have a higher probability of sharing the same parent or parents (Wang, 2009). The SA method (Wang, 2009) determines the size of the parental generation by estimating the probability that dyad relationships are either full‐ or half‐siblings in a sample from the same cohort, sharing two, one, or zero parents, respectively;

$$\frac{1}{\mathrm{Ne}}=1+\frac{3\alpha }{4}\left(Q_{1}+Q_{2}+2Q_{3}\right)‐\frac{\alpha }{2}\left(\frac{1}{N_{1}}+\frac{1}{N_{2}}\right)$$

Equation 10 from Wang (2009)

where *Q*_1,_ *Q*_2_, and *Q*_3_ are the paternal, maternal half‐sibs, and full‐sibs, respectively, *N*_1_ and *N*_2_ are the number of male and female parents, and α is a measurement of the deviation from Hardy–Weinberg proportions in genotype frequencies (Wang, 2009). The SA method was implemented in the program COLONY (Wang, 2009).

Both *Nb*(*LD*) and *Nb*(*SA*) were estimated for the EAP across four year‐of‐birth cohorts (2010, 2011, 2012, 2013) where sample size per cohort was greater than 25 individuals. Estimates of *Nb* were made using either SNP or microsatellite marker data. To estimate *Nb*(*LD*) with *NeEstimator v2.1* (Do et al., 2014), a random mating model was specified, rare alleles which upwardly bias estimates were excluded using the criterion PCrit= Pcrit0.05 as recommended in Waples and Do (2010), and jackknife confidence intervals that accouns for pseudo‐replication due to physical linkage and overlapping loci pairs were used (Jones et al., 2016; Waples & Do, 2010). To estimate *Nb*(*SA*), relatedness coefficients were estimated for individuals within each year‐of‐birth cohort using COLONY v2.0.5.6 (Jones & Wang, 2010). COLONY estimates the likelihoods of full‐, maternal half‐, and paternal half‐siblings depending on the mating system chosen in the programs settings, which may impact the final estimate of *Nb*(*SA*). We tested different COLONY parameters to determine any effects on the final estimates of *Nb*(*SA*) (Table S4.1). Results are presented for the maximum likelihood with random mating model, with male polygamy/female monogamy, no update of allele frequencies, medium sibship prior (sibship size per parent *k*=10, run for 5 replicate runs, error rate 0.001.

### 2.5 Inference of Nb/Na ratios

To calculate *Nb/Na* ratios, we used *Na* as described in Bruce et al., (2018), where *Na* is the number of adults in the population. As *C. carcharias* has a low intrinsic capacity for population increase, low fecundity, and low lifetime variance in reproductive success (Bruce, 2008), the *Na* estimates from Bruce et al. (2018) are assumed to apply to the time period corresponding to our study; *Na* = 750 with an uncertainty range 470 to 1,030 (Bruce et al., 2018). Our estimates of *Nb*(*LD*) and *Nb*(*SA*) were combined (herein *Nb*(*LD+SA*) to provide a single value of *Nb* with which to infer *Nb/Na* ratios. *Nb*(*LD*) and *Nb*(*SA*) were combined by taking the harmonic mean of the two values, weighted by the inverse of their variances as suggested in previous studies (see Waples & Do, 2010); see Appendix 1. In our study, the differences between the estimates from the LD and SA methods were not overly large, so using a combined estimate of *Nb* to determine the *Nb/Na* ratio would not change the conclusions described herein. Furthermore, in our study the SA estimate generally has a lower variance and provided a less bias and more precise estimate of *Ne* and therefore contributed more (around 2/3^rd^) to the final combined estimate. We also note that when two methods with approximately comparable performance provide an estimate of *Ne,* then the variance of the combined estimate will be smaller than for either estimate alone (Waples et al., 2016).

### 2.6 Expectations for Ne

To develop expectations for generational *Ne* in the EAP of *C. carcharias*, we use a simulation‐based approach. This route was taken as the assumptions of single‐sample genetic estimators of *Ne*, including LD and SA methods used herein, dictate that data used to make estimates represent a random sample of a population across an entire generation (Hare et al., 2011). Since the white shark is long‐lived and samples in this study were mostly juvenile or subadults, we instead characterize the expected *Nb/Ne* ratio using simulations based on published methods and parameterized using the life‐history of white shark. This indirectly allowed the inference of an expected *Nb/Ne* ratio to permit a better understanding of inbreeding and implied fitness of the population. We use both deterministic and forward‐time population simulations following methods described in Waples and Antao, (2014), to determine *Ne* and *Nb*. First, we implemented the discrete‐time, deterministic hybrid Felsenstein–Hill method for calculating *Ne* in iteroparous species (Waples et al., 2011). The model was implemented in the software *AgeNe* (Waples et al., 2011), herein *Nb*(*ageNe*), and parameterized using life‐history information from white sharks in the EAP (Supplementray Materials S7). Furthermore, since the Felsenstein–Hill method assumes the probability of reproduction is not affected by events in previous time periods, we also use forward‐time population simulations implemented in simuPOP (Peng & Kimmel, 2005), to create a single, isolated, randomly mating population to further characterize the *Nb/Ne* ratio under two intermittent‐breeding scenarios as in Waples and Antao (2014). Each simulation was parameterized using outputs from AgeNe, including total population size and stable age distribution in the population, given the specified vital rates and a specified number of offspring produced per cycle that survived to age 1 (N1), here N1 = 1,000. Each individual was represented by 100 microsatellite‐like loci, each having 10 possible allelic states, no mutation, and data were tracked for 50 reproductive cycles after a burn‐in period of 50 cycles. We forced a number of females to skip either zero, one, or two cycles of breeding (a proportion of females) hypothesized in this species (Domeier & Nasby‐Lucas, 2013; Mollet & Cailliet, 2002). Intermittent or skipped breeding occurs when sexually mature adults skip breeding opportunities (Last & Stevens, ; Shaw & Levin, 2011), in this case likely due to the costs of reproduction or prolonged gestation period in females (Bruce, 2008). We directly calculated mean (k) and variance (Vk) lifetime reproductive success, and *Ne* and *Nb* directly from simulation demographic data (not genetic data) for each reproductive cycle using Equations 1 and 2 from Waples et al. (2014), where presented values represent the arithmetic mean of *k*, *Vk* and the harmonic means of *Ne* and *Nb* calculated across 10 population replicates, herein *Ne*(*demo*), *Nb*(*demo*).

## 3 RESULTS

### 3.1 Cohort assignment

Using the relationship between TL and age, we found that one individual was born in 2005 with various years represented by the following number of individuals; 2007 (*n* = 3), 2008 (*n* = 6), 2009 (*n* = 10), 2010 (*n* = 30), 2011 (*n* = 43), 2012 (*n* = 53), 2013 (*n* = 67), 2014 (*n* = 23), 2015 (*n* = 9), and 2016 (*n* = 2). The physical size of individuals within age cohorts increased with age (Figure S5.1). The range of FL between age cohorts overlapped principally driven by heterogeneous year‐of‐capture sampling; 2010 (*n* = 30, 224 cm and 296 cm FL), 2011 (*n* = 43, 207 cm and 276 cm FL), 2012 (*n* = 52, 187 cm and 255 cm FL), and 2013 (*n* = 67, 174 cm and 268 cm FL). As low sample sizes can bias estimates of *Nb* using the methods of this study, only age cohorts containing greater than 25 samples were used (cohorts 2010, 2011, 2012 and 2013).

### 3.2 SNP and microsatellite loci data

The DArTsoft14 pipeline delivered 9,841 SNPs across 9,180 loci. The final SNP dataset after filtering consisted of 3,668 diallelic SNPs consisting of 236 EAP individuals with high‐quality SNP genotypes (Dataset‐2). Nineteen microsatellite loci were successfully genotyped across 181 EAP individuals. No evidence of null alleles or scoring errors was detected. The genotypic distribution of microsatellite genotypes per locus showed three loci did not conform to the expectations of Hardy–Weinberg equilibrium using an *a* *= 0.05* (loci Cca1419, Cca1072, CcSA2). These markers were removed from further analysis (LD method only). One locus (CcSA5) was not polymorphic (see Table S6.1) and was also excluded. Per individual, 97% had no missing loci while the remaining 3% of samples had three or less missing loci.

### 3.3 Estimates of Nb

Using SNP data, *Nb* estimates per year‐of‐birth cohort were similar between the LD and SA methods and had overlapping 95% confidence intervals (Table 1). Estimates of *Nb*(*SA*) were not sensitive to changes in model parameters such as the sibship prior, inbreeding settings, error rate, and polygamy settings (Table S4.1). This was consistent with the expectations of the SA estimator which becomes increasingly independent of the prior with increasing marker information and sample size. Although confidence intervals overlapped, estimates of *Nb*(*SA*) were generally higher than those determined from *Nb*(*LD*) across all cohorts. The 2011 cohort showed the largest difference between estimates; *Nb*(*SA*_2011_) = 344 (95% CI 204–923), *Nb*(*LD*_2011_)= 195.1 (95% CI 104–952.9).

**Table 1:** A comparison of empirical annual effective number of breeders *(Nb)* determined from genetic data (microsatellites—MSAT and single nucleotide polymorphisms—SNP) using either the linkage disequilibrium *Nb*(*LD*) or sibship assignment *Nb*(*SA*) method per year‐of‐birth cohort for the EAP of *C. carcharias*

Genetic Marker Type	Measurement	**2010**	**2011**	**2012**	**2013**
**MSAT**	***n***	**21**	**33**	**39**	**54**
	*Nb(LD)*	∞ (82.5‐∞)	263.9 (51.4‐∞)	128.7 (43.1 ‐∞)	112.6 (49.3–12934.9)
	*Nb_(SA)_*	33 (18,74)[7,56]	49 (30,84)[3,95]	51 (36–88)[5,97]	62 (41,96)[17,137]
**SNP**	***n***	**29**	**42**	**52**	**63**
	*Nb_(LD)_*	193.2 (91 ‐ ∞)	195.1 (104.2–952.9)	165.6 (104.2–359.6)	208.5 (116.4–712.7)
	*Nb_(SA)_*	271 (136–1430)[2,2]	344 (204–923)[4,4]	241 (157–399)[8,6]	289 (200–461)[8,10]
	*Nb*(*LD + SA*)(*±SD*)	^‐^	233.2(±69.5)	206.1(±45.9)	252.0(±46.7)
	*Nb/Na* ^a^	‐	0.31	0.27	0.34

Lower and upper confidence intervals in braces (lower CI‐upper CI) and the number of samples used to make the estimates, *n*, is reported. The standard deviation (±*SD*) is reported for the combined estimate of *Nb*(*LD+SA*) and the number of full‐ and half‐sibling pairs is reported in square braces [full‐sib, half‐sib in square brackets].

^a^ The ratio *Nb/Ne* determined using combined estimate, where *Na* represents the adult population size, estimated for the year 2017 (Bruce et al., 2018).

Comparing between the SA and LD method using data from microsatellite loci, estimates of *Nb*(*LD_MSAT_*) were higher than the equivalent estimate of *Nb*(SA_MSAT_). The number of estimated full‐ and half‐sibships in each cohort sample was high, and pairwise probabilities were low (data not shown) compared to those sibships estimated using SNPs. This resulted in *Nb*(*SA_MSAT_*) estimates being substantially lower than the equivalent SNP estimate, with the exception of 2011 *Nb*(*SA_MSAT_*) (Table 1). The *Nb*(*SA_MSAT_*) were the least precise estimates, where all but one cohort (2013) did not return an upper (95% CI) estimate.

The ratio *Nb/Na* was estimated using combined estimates of *Nb*(*LD+SA*). The SNP‐based *Nb* estimate for the 2010 cohort contained at least one infinite upper estimate of *Nb*, so in this case we did not calculate a combined estimate. For cohorts 2011 to 2013, *Nb*(*LD+SA*) ranged from the smallest estimated value in 2012, *Nb*(*LD+SA_2012_*) (*45.9 SD*) to the largest in 2013, *Nb*(*LD+SA_2013_*)*=252* (*46.7 SD*) (Table 1). The inferred ratio of *Nb/Na* ranged from 0.27 to 0.34; *Nb/Na*_*2012*_ = 0.27 (0.44–0.2) to *Nb/Na*_*2013*_= 0.34 (0.54–0.24). The intervals (in parentheses) were calculated using the lower and upper uncertainty estimates of *Na* from Bruce et al., (2018).

The ratio of Nb/Ne was evaluated using simulations. Using a standard model implemented in *AgeNe* yielded estimates of *Nb*(*demo*), *Ne*(*demo*) of 372.7 and 857.2 respectively, and an *Nb*(*demo*)*/Ne*(*demo*) ratio of 0.43. To account for variations in breeding biology, further forward‐time population simulations in SimuPOP showed the equivalent no‐skip breeding model closely reflected AgeNe results (*Nb*_*demo*_ = 365.46, *Ne* *_demo_* = 860.67), validating the model, while alternate breeding models decreased the *Nb/Ne* ratio (see Table S7.2).

## 4 DISCUSSION

Monitoring *Ne* can inform management decisions in populations of conservation concern, where *Nb* is analogous to *Ne* except that it represents the effective number of breeders per year rather than per generation. Using data from SNP and microsatellite loci and two single‐sample genetic estimators of effective popultion size, our results show the effective breeding population (*Nb*) of the EAP has remained unchanged across four successive years (2010–2013), although we caution that these results may not be indicative of a broader temporal trend. Our study supports existing evidence (Hillary et al., 2018; Roff et al., 2018) that the white shark population has not changed significantly in size over the years studied herein, despite measures implemented to rebuild the population. The white shark recorded substantial declines through the 20^th^ century in Australia and New Zealand and has since been the subject of legislated protection and management interventions targeted toward population recovery (i.e., National Plans of Action for the Conservation and Management of Sharks Commonwealth of Australia, 2013; EPBC, 1999; Shark Advisory Group, 2004). However, monitoring *Nb* using the methods describe herein could assist management and conservation efforts. Indeed, as past studies have shown, monitoring *Nb* for as few as five consecutive reproductive cycles could be used to detect change in *Nb* (declines), even in species with long generation intervals (Antao et al.,^2011^; Wang, 2005; Luikart et al., 2020) such as the white shark, with implications for both *N* and *Ne* in some circumstances. For example, if *Nb* were to decline significantly for multiple reproductive cycles, then both *Ne* and *Nc* may be affected (Luikart et al., 2020).Alternatively, if *Nb/N* ratios are observed to be stable over many generations, then it has been suggested that *N* (or *Na*) may be inferred from *Nb*, which would be of use to population monitoring and evaluation of conservation and management actions (Luikart et al., 2020). To this end, we recommend using *Nb* to track year‐to‐year changes in the effective number of breeders as a timely assessment of population status over time to provide insights into the effects of current management actions and co‐occurrences such as environmental changes. As in this study, future tissue samples for *Nb* monitoring could be obtained as part of existing bather protection programs (i.e., SMART drumlines; see Tate et al., 2019).

In this study, we used two genetic marker types (SNPs and microsatellites) and two single‐sample genetic estimators of effective population size (LD and SA) to estimate *Nb*. Both estimators showed more precision and power when SNPs were used to estimate *Nb* compared to the few microsatellites used in this study. We therefore recommend the use of SNPs for the future monitoring of the EAP. Although *Nb*(*LD*) estimated from both SNP and microsatellite were comparable and results reflected differences between genetic marker type similar to those reported in previous studies (e.g., Beebee, 2009), here the few microsatellite loci used were unable to estimate upper CIs for age cohorts without significant sampling effort (>50 samples). Of note, estimates of *Nb*(*LD_MSAT_*) were consistently higher than the equivalent estimates of $Nb({\mathrm{SA}}_{\mathrm{MSAT}})$. This can be attributed to the overestimation of sibship dyads, which can be expected to decrease estimates of *Nb* (Table 1). This has been noted in previous studies (Ackerman et al., 2017; Wang, 2009) which have demonstrated that false sibships (type I errors) occur with a higher frequency compared to false nonsibships (type II errors) when either genetic information or true sibship within a sample is insufficient (i.e., few loci, low polymorphism, small sample size relative to total population size, low inclusion of siblings). *Nb* estimated using SNPs differed between methods such that *Nb*(*LD*) was lower compared to *Nb*(*SA*), although differences were not significant having overlapping CIs. *Nb*(*LD*) estimated using SNPs showed those cohorts with larger numbers of samples (i.e., 2013) provided more precise estimates, a result expected given genetic methods for estimating contemporary effective size depend on signals that are proportional to *1/Ne* (Waples et al., 2014, 2018).

Monitoring studies are often focused on the number of individuals in a population; however, the relationship between effective size and population size (i.e., *Ne/Na, Nb/N*) is important to understand since genetic drift results in the loss of neutral genetic variation at a rate rate inversely proportional to *Ne* per generation, not *N* (Wright, 1931) and can therefore be useful for examining how different ecological factors influence genetic variation (Nunney, 1996). In this study, the ratio of *Nb/Na* was approximately 1/3 of mature adults for a single reproductive cycle, where mature adults represent different adult age classes. This is somewhat comparable to ratios inferred for other Carcharhinidae, including *C. plumbeus* (sandbar shark) in Delaware Bay, North Atlantic, which ranges between 0.50 (95% CI 0.45) and 0.63(95% CI 0.57) (Portnoy et al., 2009). Since a ratio of *Nb/Na* applies to a single reproductive cycle; when ratios are close to 1, we can infer that the majority of the adult population contribute to the next generation and that the offspring number per adult approaches the standard scenario of binomial distribution (Hedgecock, 1994). In contrast, when ratios are < 1, we can infer there is some deviation from the ideal (Hare et al., 2011). A number of factors will affect this relationship (*Ne/Na, Nb/Na*) including fluctuations in population size and several important life‐history factors that change variance in reproductive success (e.g. mating system, generation time, sex difference including sex ratio, survival, recruitment age). In one case, *Nb* may be expected to be reduced relative to *Na* if females with high fecundity skip reproductive cycles after giving birth, resulting in different females breeding in different cycles (Waples & Anato, 2014). This should decrease both lifetime *Vk* and *Nb*, while increasing *Ne*. The ratio reported herein appears to be consistent with expectations for the breeding behavior of *C. carcharias*, suspected to undergo intermittent breeding (Bruce, 2008). Observations suggest the gestation period of *C. carcharias* females may approach 18 months from fertilization to parturition (Bruce, 2008; Mollet et al., 2000), resulting in the unavailability of a portion of adult females to produce offspring each cycle. However, we caveat that *Nb/Na* ratios determined in this study used estimates of *Na* from best available information from close‐kin‐mark‐recapture estimates for the EAP in 2017 (see Bruce et al., 2018). Thus we assumed temporal stability of *N* over time; an assumption which would be violated if *N* has increased (or decreased) over the time period to which our *Nb* estimates apply (2010‐2013).

Since neutral genetic variation is lost at a rate of 1/2*Ne* per generation (Wright, 1931), even numerically large populations can be at genetic risk if *Ne* is small (Waples et al., 2018). Although important, due to sampling restriction (i.e., difficulty sampling across a generation as required by estimators) and uncertainty of breeding histories, we could not estimate *Ne* directly nor did we consider the linear relationship between *Nb* and *Ne* which requires either true or estimated *Nb/Ne* to be quantified (Waples et al., 2013). Blower et al., (2012) provided the first genetic estimate of *Ne* for the EAP using 6 SSR markers. Finite point‐estimates showed effective population size was *Ne* = 380, Pcrit = 0.18 (95% CI = 31 − ∞, n = 62), however, as the authors used both juvenile (n = 55) samples and adult samples (n = 7), this value likely represents something between *Nb* and *Ne*. Instead in this study, using simulations, we show that *Nb* maybe expected to be*less than Ne* using life‐history information for white shark, and that *Nb* can be expected to be much less than *Ne* if intermittent breeding were occurring. This aligns with expectations of *Ne* where a small number of offspring, delayed maturation, intermittent breeding, and low lifetime variance in fecundity act to increase *Ne* relative to *Nb* or *N* (Waples & Antao, 2014). This result is important as it suggests the study population in the EAP at least exceeds the inbreeding avoidance goal (*Nb*100) (Frankham et al., 2014). *Ne* > 100 (previously the *Ne‐50* *rule*) describes the short term goal required to avoid inbreeding, which results in excess homozygosity for deleterious and recessive alleles, leading to inbreeding depression and reduced fitness (Frankham et al., 2014). However, in relation to the long‐term viable population benchmark, *Ne* 1,000 (previously *Ne* > 500) (Frankham et al., 2014) our results are less certain, and we caveat that this rule (Ne > 1000) refers to the loss of additive genetic variation that may negatively effect adaptation in response to changes in selective regimes, not inbreeding effective size as estimated herein. We suggest any genetic effects of a recently and significantly reduced population size in the EAP, such as a decline in *Ne* or loss of heterozygosity, may not be fully realized until adequate benchmark studies can be completed (i.e., historical or ancient DNA). However, genetic bottlenecks in white sharks have been recorded elsewhere (O’Leary et al., 2015). Given this, together with the lack of evidence from other studies to date of an expected recovery (except see Department of Primary Industries, 2019), our results emphasize the importance of continued monitoring, improved protections, and interventions to reduce mortality. Indeed, the vulnerability of chondrichthyan fishes to exploitation has been comprehensively documented (Hutchings et al., 2012) and relative to other marine fish, the intrinsic capacity for population increase and rebound potential in white shark is low (Cortés, 2002) (i.e., long‐lived, late age to maturity, high juvenile survival). In addition, shark species often travel large distances and use different habitats throughout their lives (Fujioka & Halpin, 2014), where they may be vulnerable to environmental changes (density, food availability, climate, illegal fishing). Regrettably, mortalities continue to occur in the EAP driven by action taken to mitigate human–shark interactions. During the years 2018–2019, 51 bather protection nets were distributed across seven regions of NSW (Australia). Catches of white shark and other shark species are only recently increasing year‐on‐year (Department of Primary Industries, 2019) following long term declines over 80 years of the bather‐protection program along the east coast of Australia, which has been lethal for sharks despite catch‐and‐release programs (Roff et al., 2018). The recent modeling of the recovery of the North West Atlantic white shark population provides a useful principal in this regard; “every fish counts” (Bowlby & Gibson, 2020, p.9).

## 5 CONCLUSION

We have used genetic data to estimate the size of the effective breeding population (*Nb*) over four consecutive years (2010 to 2013) for white sharks in an east Australian–New Zealand population, representing an indirect measure of reproductive effort over a relatively short temporal period. Our results suggest Nb has remained stable over four years and agrees with previous studies that report stability of population size in the EAP, where *Nb* estimates were more precise using data from SNP rather than microsatellite loci and estimates from two single‐sample genetic estimators were similar. However, longer time series of data are needed to determine the efficacy of past and present management and conservation actions on the genetic constitution of the population. We suggest future monitoring using *Nb* should continue given the availability of nonlethal tissue samples from bather protection programs, the ease of genomic data collection and analyses, and the complementary nature of *Nb* and *Na* estimates.

## CONFLICTS OF INTEREST

This project was funded by the New South Wales Department of Primary Industries, Australia. The authors have no conflicts of interest to declare.

## AUTHOR CONTRIBUTION

**Danielle Louise Davenport:** Conceptualization (equal); Formal analysis (lead); Methodology (lead); Project administration (lead); Visualization (lead); Writing‐original draft (lead); Writing‐review & editing (lead). **Paul Butcher:** Conceptualization (equal); Funding acquisition (lead); Resources (equal); Supervision (equal); Writing‐original draft (equal); Writing‐review & editing (equal). **Sara Andreotti:** Formal analysis (supporting); Writing‐original draft (supporting); Writing‐review & editing (supporting). **Conrad A. Matthee:** Writing‐original draft (supporting); Writing‐review & editing (supporting). **Andrew Jones:** Formal analysis (equal); Methodology (equal); Writing‐original draft (equal); Writing‐review & editing (equal). **Jennifer Robyn Ovenden:** Conceptualization (equal); Formal analysis (supporting); Methodology (equal); Project administration (equal); Supervision (lead); Writing‐original draft (equal); Writing‐review & editing (equal).

## Animal studies

NSW DPI provided scientific (Ref. P01/0059(A)), Marine Parks (Ref. P16/0145‐1.1), and Animal Care and Ethics (ACEC Ref. 07/08) permits.

## Supporting information

Appendix S1–S6

## Data Availability

Data for this study are available at UQ espace: https://doi.org/10.14264/uql.2020.634. All plots in this study were made using the *ggplot2* package in R (Wickham, 2016).

## Appendix 1

### Inverse‐variance weighted mean method and worked example

This is a worked example using data from the cohort 2013. This weighted mean will give the lowest variance of any weighted mean of the values. As with nearly all Ne calculations the harmonic mean must be used as the real quantities of interest are proportional to 1/*Ne*.

**Values**

**LDNe: 208.5**

**COLONY: 289**

**Sample Size: 63**

#### The Variances

Unfortunately, neither COLONY or NeEstimator (Do et al., 2014; Jones & Wang, 2010) provides the raw variance figures required; however, these can be approximated by working backwards from the provided confidence intervals.

#### COLONY

Colony generates 95% confidence intervals using the following formula [cite]:

$$CI:\frac{1}{2N_{e}}\pm 1.96\sqrt{V^{*}},$$

where V^*^ is the variance of the estimate of 1/$2N_{e}$. Knowing the upper and lower bounds of this confidence interval, we can estimate V^*^ as,

$$V^{*}={\left[\frac{2L^{*}‐1\;/\;N_{e})}{3.92}\right]}^{2},$$

or,

$$V^{*}\approx {\left[\frac{1\;/\;L)‐1\;/\;N_{e})}{4}\right]}^{2}.$$

where L^*^ and L are the lower confidence bounds in terms of 1/$2N_{e}$ and $N_{e}$ respectively. An identical argument follows for the upper bounds. However, we desire the variance of $N_{e}$, which we can approximate using a first order Taylor expansion. That is, *Var*[*f*(*x*)] ≈ (*f*′*E*[*X*])^2^ *Var*[*X*]. Substituting in our particular case,

$$Var\left[N_{e}\right]\approx {{\left[\frac{‐4}{2}{N_{e}}^{2}\right]}^{2}V}^{*},$$

$$Var\left[N_{e}\right]\approx {\left[\frac{‐4}{2}{N_{e}}^{2}\right]}^{2}{\left[\frac{1\;/\;L)‐1\;/\;N_{e})}{4}\right]}^{2},$$

$$Var\left[N_{e}\right]\approx \frac{1}{4}{\left[N_{e}\right]}^{4}{\left[1\;/\;L)‐1\;/\;N_{e})\right]}^{2}.$$

We also have

$$Var\left[N_{e}\right]\approx \frac{1}{4}{\left[N_{e}\right]}^{4}{\left[1\;/\;U)‐1\;/\;N_{e})\right]}^{2}$$

via the upper bound. These are now in terms of known values and we can estimate $Var\left[N_{e}\right]$.

**Table:**

*Ne*	*U*	*L*	$Var\left[N_{e}\right]$.
289	–	200	4,134.811506
289	461	–	2,906.636596
Mean			3,520.724051

### NE ESTIMATOR

Similar to COLONY Ne Estimator does not provide raw variances, and we need to work in terms of the confidence intervals for $\left({\hat{r}}^{2}‐drift\right)$ which we will call here *r**. The drift term is ~ 1/*S*, where S is the sample size. The 95% confidence interval for *r** is explicitly normal in the case of the jackknife confidence interval (Jones et al., 2016). Thus, *V**, the variance of r^*^ is approximated by

$$V^{*}\approx {\left[\frac{U^{*}‐r^{*}}{2}\right]}^{2}\approx {\left[\frac{L^{*}‐r^{*}}{2}\right]}^{2},$$

where U and L are the upper and lower bounds provided for r^*^. However, again, we wish to have the variance in terms of $N_{e}$. Using the same approach as for COLONY, we will approximate this using Var[*f*(*x*)] ≈ (*f*′*E*[*X*])^2^Var[*X*]. The true function used to calculate N_e_ using r^*^ can be found in Table 1 in Waples and Do (2008), here we use the simpler original form (Equation 1 in Jones et al., 2016 and others) as our $f\left(X\right)$ for this estimate, that is

$$N_{e}=1\;/\;3({\hat{r}}^{2}‐drift))=1\;/\;3(r^{*})).$$

Working as before,

$$Var\left[N_{e}\right]\approx {{\left[\frac{‐1}{3{\left(r^{*}\right)}^{2}}\right]}^{2}V}^{*}$$

$$Var\left[N_{e}\right]\approx \frac{1}{4}\frac{1}{\left[{\left(r^{*}\right)}^{4}\right]}\frac{1}{9}{\left(L^{*}‐r^{*}\right)}^{2}$$

$$Var\left[N_{e}\right]\approx \frac{1}{36}\frac{1}{\left[{\left(r^{*}\right)}^{4}\right]}{\left(L^{*}‐r^{*}\right)}^{2}\approx \frac{1}{36}\frac{1}{\left[{\left(r^{*}\right)}^{4}\right]}{\left(U^{*}‐r^{*}\right)}^{2}$$

However, we still need to obtain $U^{*}$ and $L^{*}$ the upper and lower confidence bounds for *r**. This can be achieved by inverting the function of r^*^ and S from Table 1 in Waples and Do (2008). In this particular case, we cannot use the simple approximation. In our case, the inverted function is

$$r^{*}=\frac{‐69S^{2}+\sqrt{10000S^{4}N_{e}^{2}+4761S^{4}‐248400S^{3}N_{e}}+1800SN_{e}^{2}}{1800S^{2}N_{e}^{2}}‐1\;/\;S).$$

**Table:**

N_e_	CI Bound (N_e_)	CI Bound ($r^{*}$)	$Var\left[N_{e}\right]$
208.5	116.4	0.000368638	6,523.337897
208.5	712.7	6.05919E−05	10,252.64749
Mean			8,387.992693

### WEIGHTED MEAN AND FINAL VARIANCE

Now we follow the formula for the weighted harmonic mean,

$$\overset{‐}{x}=\frac{1}{\sum_{i}w_{i}\frac{1}{x_{i}}},$$

where the weights, $w_{i}$, sum to 1. In this case, we need to normalize the inverse variances to sum to 1.

**Table:**

Ne	Method	Variance	Inverse variance	Normalized inverse‐variance weight	$\frac{1}{N_{e}}$
289	COLONY	3,520.724051	0.000284032	0.704357394	0.003460208
208.5	LD	8,387.992693	0.000119218	0.295642606	0.004796163

The final mean estimate is:

$\frac{1}{\left(0.704357394∙0.003460208\right)+\left(0.295642606∙0.004796163\right)}=259.3917339$

The COLONY estimate has a lower variance and thus contributes around 2/3 of the final estimate (70.4%).
