# Correction to Effective number of white shark (*Carcharodon carcharias*, Linnaeus) breeders is stable over four successive years in the population adjacent to eastern Australia and New Zealand

**DOI:** 10.1002/ece3.70221

**Published:** 2024-09-18

Davenport, D., Butcher, P., Andreotti, S., Matthee, C., Jones, A., & Ovenden, J. (2021). Effective number of white shark (*Carcharodon carcharias*, Linnaeus) breeders is stable over four successive years in the population adjacent to eastern Australia and New Zealand. *Ecology and Evolution*, ***11***, 186–198.

In our study, we made estimates for the effective number of breeders (Nb), an analogue of effective population size (Ne), in a population of white sharks. Due to an identified error in an intermediate data file used to make Nb estimates, we re‐completed the analysis from the raw data and re‐made the affected intermediate data file.

Because of this error the following text was affected in the Abstract:

Where the text previously stated “… the 2013 age cohort was 289 (95% CI 200–461) and Nb (LD) was 208.5 (95% CI 116.4–712.7). We show that over the time period studied, Nb was stable and ranged between 206.1 (SD ± 45.9) and 252.0 (SD ± 46.7) per year using a combined estimate of Nb(LD+SA) from SNP loci.” It should instead read “… the 2013 age‐cohort Nb(SA) was 174 (123, 252 95% CI) and Nb(LD) was 179.8 (121.9, 320.8 95% CI). We show that over the time period studied *Nb* was stable (showed no significant detectable trend), and ranged between 74.6 (20.6 ± SD) and 228.7 (57.8 ± SD) per year using a combined estimate of Nb(LD+SA) from SNP loci.”

When correcting the result presented here, changes were made to the Materials and Methods where the line previously read: “Postprocessing of SNPs was completed in R (R Core Team, 2018) using the R‐Package radiator 0.0.5 (Gosselin, 2017) and custom R‐scripts following current best practice (O'Leary et al., 2018; Shafer et al., 2017)”, we wish to state: “Post‐processing of SNPs was completed in R (R Core Team, 2018) using various R‐packages (dartR v.2 (Mijangos et al., 2022), hieferstat (Goudet & Jombart, 2022))”. We would also like to add “We use the method of Luikart et al., (2020) (ordinary least squares regression), and Generalized least‐squares (GLS regression) that incorporates an estimate of autocorrelation, to test the significance of a trend in Nb estimates across cohorts”.

In the Results section, the text in Table 1 reads:

**TABLE 1:** A comparison of empirical annual effective number of breeders (Nb) determined from genetic data (microsatellites—MSAT and single nucleotide polymorphisms—SNP) using either the linkage disequilibrium Nb(LD) or sibship assignment Nb(SA) method per year‐of‐birth cohort for the EAP of *C. carcharias* ^*^.

Type	Measurement	2010	2011	2012	2013
**MSAT**	***n***	**21**	**33**	**39**	**54**
	${\mathrm{Nb}}_{\mathrm{LD}}$	∞ (82.5–∞)	263.9 (51.4–∞)	128.7 (43.1−∞)	112.6 (49.3–12934.9)
	${\mathrm{Nb}}_{\mathrm{SA}}$	33 (18,74)[7,56]	49 (30,84)[3,95]	51 (36–88)[5,97]	62 (41,96)[17,137]
**SNP**	***n***	**29**	**42**	**52**	**63**
	${\mathrm{Nb}}_{\mathrm{LD}}$	193.2 (91 ‐ ∞)	195.1 (104.2–952.9)	165.6 (104.2–359.6)	208.5 (116.4–712.7)
	${\mathrm{Nb}}_{\mathrm{SA}}$	271 (136–1430)[2,2]	344 (204–923)[4,4]	241 (157–399)[8,6]	289 (200–461)[8,10]
	${\mathrm{Nb}}_{\mathrm{LD}}+{\mathrm{Nb}}_{\mathrm{SA}\;\left(\pm \mathrm{SA}\right)}$	–	233.2(±69.5)	206.1(±45.9)	252.0(±46.7)
	Nb/Na ^†^		0.31	0.27	0.34

We wish to correct Table 1 to the following:

**Table:** 

Type	Measurement	2010	2011	2012	2013
**MSAT**	***n***	**21**	**33**	**39**	**54**
	${\mathrm{Nb}}_{\mathrm{LD}}$	∞ (82.5–∞)	263.9 (51.4–∞)	128.7 (43.1–∞)	122.6 (49.3, 12,934.9)
	${\mathrm{Nb}}_{\mathrm{SA}}$	33 (18,74)[7,56]	49 (30,84)[3,95]	51 (36,88)[5,97]	62 (41,96)[17,137]
**SNP**	***n***	**29**	**39**	**52**	**63**
	${\mathrm{Nb}}_{\mathrm{LD}}$	62.1 (32.7, 249.5)	208.1 (110.1, 1135.9)	201.9 (126.7, 449.0)	179.8 (121.9, 320.8)
	${\mathrm{Nb}}_{\mathrm{SA}}$	96(56,198) [7,3]	247(148,567) [4,5]	196(132,332) [8,15]	174(123,252) [16,22]
	${\mathrm{Nb}}_{\mathrm{LD}}+{\mathrm{Nb}}_{\mathrm{SA}\;\left(\pm SD\right)}$	74.6 (20.6)	228.7 (57.8)	197.21 (39.08)	174.85 (29.3)
	Nb/Na ^†^	0.10	0.30	0.26	0.23

*Lower and upper confidence intervals in braces (lower CI‐upper CI) and the number of samples used to make the estimates, *n*, is reported. The standard deviation (±*SD*) is reported for the combined estimate of *Nb*(*LD+SA*) and the number of full‐ and half‐sibling pairs is reported in square braces [full‐sib, half‐sib in square brackets].

^†^The ratio Nb/Na was determined using combined estimate, where *Na* represents the adult population size, estimated for the year 2017 (Bruce et al., 2018).

In the results where the line previously stated “The final SNP dataset after filtering consisted of 3,668 diallelic SNPs consisting of 236 EAP individuals with high‐quality SNP genotypes (Dataset‐2).” It should read: “The final SNP dataset after filtering consisted of 4256 diallelic SNPs consisting of 235 EAP individuals with high‐quality SNP genotypes (Dataset‐2)”.

The line in the results section which states “For cohorts 2011 to 2013, Nb(LD+SA) ranged from the smallest estimated value in 2012, Nb(LD+SA2012) (45.9 SD) to the largest in 2013, Nb(LD+SA2013) = 252 (46.7 SD) (Table 1). The inferred ratio of Nb/Na ranged from 0.27 to 0.34; Nb/Na2012 = 0.27 (0.44–0.2) to Nb/Na2013 = 0.34 (0.54–0.24)" should instead read “For cohorts 2010 to 2013, $\mathrm{Nb}\left(\mathrm{LD}+\mathrm{SA}\right)$ ranged from the smallest estimated value in 2010, $\mathrm{Nb}\left(\mathrm{LD}+{\mathrm{SA}}_{2010}\right)=74.6\left(20.6\;\mathrm{SD}\right),$ to the largest in 2011, $\mathrm{Nb}\left(\mathrm{LD}+{\mathrm{SA}}_{2011}\right)=228.7\left(57.8\;\mathrm{SD}\right)$ (Table 1). The inferred ratio of Nb/Na ranged from $\mathrm{Nb}/{\mathrm{Na}}_{2010}$ = 0.10 to $\mathrm{Nb}/{\mathrm{Na}}_{2011}$ = 0.30, calculated using estimates of Na from Bruce et al. (2018).”

We wish to state in the results that: “Tests for a trend in Nb overtime using regression methods were not significant (OLS regression *p*‐value = 0.345; GLS regression *p*‐value = 0.861)”.

In the discussion, where the text states “Nb estimated using SNPs differed between methods, such that Nb(LD) was lower compared to Nb(SA)”, it should instead read “Nb estimated using SNPs differed between methods, although differences were not significant and method estimates have overlapping CIs”.

We wish to acknowledge Dean Blower and Paul Butcher who identified an error in the original published version of this manuscript. We thank Dean Blower for his extensive analysis and review of the subsequent correction.

Corrected data inputs and analysis for this study are available at zendoo https://doi.org/10.5281/zenodo.10172611.

We apologize for this error.

