# Supplementary material for: Effective number of white shark (Carcharodon carcharias, Linnaeus) breeders is stable over four successive years in the population adjacent to eastern Australia and New Zealand
Source: Ecol Evol. 2020 Dec 9;11(1):186–98. doi: 10.1002/ece3.7007 (PMC7790646; doi:10.1002/ece3.7007)
Supplement: Supplementary file 1 — Appendix S1–S6 [file ECE3-11-186-s001.docx]

Supplementary Materials Davenport et al., 2020

Effective number of white shark (*Carcharodon carcharias,* Linneaus) breeders is stable over four successive years in the population adjacent to eastern Australia and New Zealand

Table of Contents

[Supplementary Appendix S1 2](#_Toc52875776)

[Supplementary Appendix S2 3](#_Toc52875777)

[Supplementary Appendix S3 7](#_Toc52875778)

[Supplementary Appendix S4 10](#_Toc52875779)

[Supplementary Appendix S5 11](#_Toc52875780)

[Supplementary Appendix S6 12](#_Toc52875781)

[References 13](#_Toc52875782)

# Supplementary Appendix S1

**Table S1.** Samples number (N) and collection locations (not including duplicates of samples included for quality control during sequencing)

| **Sample Collection Location** | **N** |
| --- | --- |
| East Australia | 247 |
| South Africa, Western Cape | 20 |
| West Australia | 3 |
| South Australia | 9 |
| Total | 279 |

# Supplementary Appendix S2

Age in sharks is most commonly achieved by counting growth rings in vertebrae, fin spines or other hard parts( Cailliet, Smith, Mollet, & Goldman, 2006)Together with a measurement of body length, size-at-age can be used to model the relationship between the two, where the Bertalanffy growth function (VBGF) (Von Bertalanffy, 1938) is the most commonly used for species of shark (Smart, Chin, Tobin, & Simpfendorfer, 2016). To estimate the age of each sample and assign each to a year-of-birth cohort, a three-parameter generalised VBGF was used following the recommendations of Pardo et al. (2013) to transform the relationship of total length (TL) to relationship at age:

$$E[L|t] = L\infty-(1-e^{-K(t-to)})$$

(Cailliet et al., 2006)

where E[L|t] is the expected or average TL at time (or age) *t*, L∞ is the asymptotic average length, K is the Brody growth rate coefficient (units are yr^-1^ ), the third parameter (*t*_0_) is a modelling artefact representing time or age when the average length is 0. Since there is considerable variability in published growth parameters for white sharks which vary between region and sex (Cailliet, Natanson, Welden, & Ebert, 1985; Tanaka, Kitamura, Mochizuki, & Kofuji, 2011; Wintner, 1999), we used specific growth parameters for white sharks in east Australia found in O’Connor (O’Connor, 2011). The parameters L∞ = 7.98.94 cm TL (male) and L∞ = 7.19 cm TL (female), k = 0.047 y-1 (male) and k = 0.056 y-1 (females) and t0 = -3.8 (both sexes) were used in this study, as defined in Table 2.2 of O’Connor (2011).

# Supplementary Appendix S3

A two-stage filtering approach was employed to maximise the number of SNP markers remained to estimate Nb. Firstly, we use all SNPs (9841 SNPs across 9180 loci) and all samples (East Australia *n* = 247 Western Australia *n* = 3; South Australia *n* = 9; South Africa *n* = 20; total *n* = 279). We call this Dataset-1. We filter this data using the R-Package *radiator 0.0.5* (Gosselin, 2017) specifying the values outlined in Table S3.1.

Following initial filtering (removing possible genotyping errors) we used Dataset-1 to perform tests for divergent individuals. Discriminant analysis of principal components (DAPC) (Jombart, Devillard, & Balloux, 2010) was used to investigate the genetic similarity of collected samples. DAPC is a multivariate method used to identify and describe clusters of genetically related individuals. The genetic variation of samples is partitioned into two components: variation between groups and within groups, and it maximizes the former resulting in linear combinations of alleles which best separate the clusters. Alleles that most contribute to this discrimination are therefore those that are the most markedly different across groups, and allows the identification of samples which may be genetic divergent. DAPC was used as implemented in the R-package *adegenet* (Jombart et al., 2010). The optimal number of discriminant functions to retain was calculated using the function adegent::xvalDAPC using 80% of the data in the training set, and the number of PCs retained in the final DAPC were associated with the lowest Mean Squared Error. As indicated in Figure S3.1 below, two samples collected from east Australia appeared distinct from other east Australia samples. These samples were removed from subsequent analysis 6) We also used Dataset-1 to perform tests for outlier loci (under selection, non-neutral). We used  *pcadapt* (Luu, Bazin, & Blum, 2017) in R which performs genome scans to detect genes under selection. We used K principal components, K = 3 and removed loci with q-values smaller than  a = 0.05 (false discovery rate). We identified and removed 30 potential outlier loci using this method.

Following these initial steps, we kept only individuals thought to be of EAP origin (from the NSW DAPC cluster, see Figure S3.1) and all SNPs originally genotyped in these samples (9180 / 9841 SNP/Loci). We call this Dataset-2. We then filter Dataset-2 following the steps outlined in Table S3.2. We use this dataset to make estimates of Nb in our study.

**
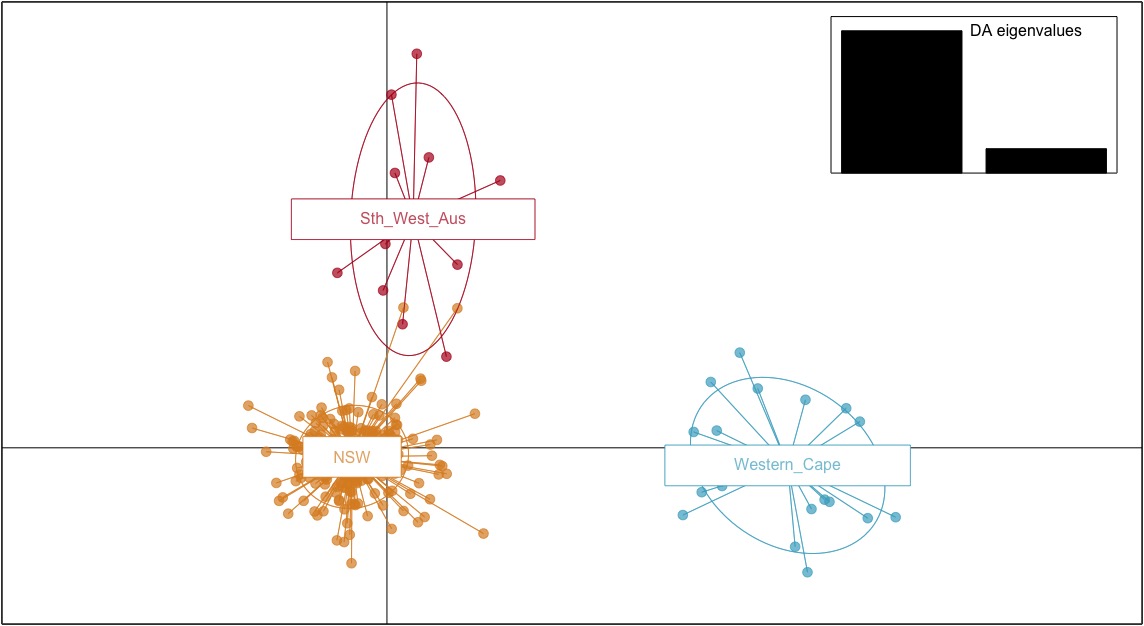
**

**Figure S3.1** DAPC (x= principle component 1, y = principle components 2) using all samples included in the study.

**Table S3.1** Filtering of 9180/9841 DArT loci/SNP using all samples (n = 279) Dataset 1

| **Filter** | **Loci/SNP** | **Samples Removed** | **Notes** |
| --- | --- | --- | --- |
| Initial | 9180/9841 | 3 | 3 samples poorly genotyped |
| Reproducibility > 95% | 8899/9525 |  | A measure of marker quality through technical replication |
| Call rate >= 75% | 8620/9122 |  | Proportion of samples for which the marker is scored |
| Coverage (Min 5, Max 25) | NA/7702 |  | A maximum read depth equal to $d+4*\sqrt{d}$, where d is the average read depth. This step reduces the number of false heterozygotes due to sequencing errors or due to the presence of paralogs (Li, 2014) |
| Individuals missing > 80% | 1 | 1 |  |
| Markers missing > 70% | NA/7618 |  |  |
| Duplicated genomes | 8 | 8 | Duplicates included across each of the 3x 96-well plates |
| Monomorphic | 7212/7599 |  |  |
| HWD Exact Test midp <0.01 in at least 3/3 groups | 7592/7207 |  |  |

**Table S3.2** Filtering EAP samples (N = 247+2 EA Duplicates), final dataset used for Nb estimates – Dataset 2

| **Filter** | **Loci/SNP remaining** | **Samples Removed/**  **Samples Remaining** | | **Notes** | |
| --- | --- | --- | --- | --- | --- |
| Initial | 9180 / 9841 | 247 |  | | |
| Genotyping Quality |  | 3/244 | 3 samples poorly genotyped | | |
| Divergent Individuals identified using DAPC (Dataset 1as above) | 2 individuals removed | 2/242 | Within-group structure, biases genetic Ne estimates due to the Wahlund effect (Wahlund, [1928](https://onlinelibrary.wiley.com/doi/10.1111/eva.12551#eva12551-bib-0054)) | | |
| Reproducibility > 98% | 6449 / 6850 |  | | |  |
| Monomorphic | 6416 / 6815 |  | | |  |
| Minor Allele Count (MAC) > 3 | 5737 / 6083 |  |  | | |
| Coverage (Min 5, Max 25) | 5696 / 6031 |  |  | | |
| Genotyping/Call Rate | 5200 / 5420 |  |  | | |
| 1 SNP per loci (short LD) | 5014 / 5014 |  |  | | |
| Duplicated, Missing Data > 20%, or Genetically Identical Individuals (genetic distance close to 1) |  | 6/236 | Duplicates included across 3x 96-well plates – 2 known duplicates not included in count; where genetically identical or close individuals were thought to be un-identified duplicates of the same sample. | | |
| HWE Mid-p 0.1 & Outlier Loci identified in pcadapt analysis above | 3736 |  |  | | |
| FIS < - 0.5 or > 0.5 | 3668 |  |  | | |
| **Final** | **3668** | **236** |  | | |

# Supplementary Appendix S4

Here we outline the parameters used in COLONY. Multiple paternity has been documented in 12 of the 15 species (80%) of elasmobranchs including 14 species of sharks (Fitzpatrick, Kempster, Daly-Engel, Collin, & Evans, 2012; Holmes et al., 2018). In *C. carcharias* multiple paternity has been identified in one case where 8 pups from the same gravid mother were genotyped using seven microsatellite markers (Gubili, 2008). Therefore, we tested (a) female monogamy but male polygamy - allowing for full-sib relationships and paternal half-sib relationships (Jones & Wang, 2004) and (b) both female and male polygamy - allowing for full-sib relationships and both maternal and paternal half-sib relationships (Jones & Wang, 2004). We also tested the effect of either a “medium” and “weak” and “none” sib-ship prior (reduces false assignment of sib-ship) where the mean number of offspring per parent $(k)$was set for each parent where required. Here, we tested $k=10$ since litter sizes in white shark are thought to be as low as 2 and as high as 10 (Domeier, 2012).

$Nb(SA)$ was also determined using either allele frequencies calculated from all samples, or unknown allele frequency, and either inbreeding or no inbreeding. All other default parameter settings were used; diploid, a single run of medium length, medium likelihood precision, and no update of allele frequencies. The probabilities of a male or female parent being included in the candidates were set as zero as no reproductively mature sharks were sampled in this study. Results were not different between tested scenarios, see Table S3.1.

**Table S4.1** Results of $Nb(sib)$ estimated for the EAP using SNPs under different settings in COLONY implementing the Full Likelihood Assuming Random Mating method. Some cohorts are skipped due to running times and similar results observed between cohorts which were tested.

| **COLONY Parameters** | **Values** | **Cohort** | **Full-Sibs** | **Half-Sibs** | **Nb (95% CI)** |
| --- | --- | --- | --- | --- | --- |
| Update Allele Frequencies  Inbreeding  Polygamy Males/Females  Scale Full Sib-ship  Sib-ship Prior  Unknown/Known population allele freq | 0  0  0 1  1  1 10 10  1 |  |  |  |  |
|  |  | 2010 | 2 | 2 | 271 (142,1645) |
|  |  | 2011 | 4 | 4 | 344 (204,920) |
|  |  | 2012 | 8 | 6 | 241(157,415) |
|  |  | 2013 | 8 | 10 | 289 (196,474) |
| Update Allele Frequencies  Inbreeding  Polygamy Males/Females  Scale Full Sib-ship  Sib-ship Prior  Unknown/Known population allele freq | 0  0  0 1  1  3 10 10  1 |  |  |  |  |
|  |  | 2010 | 2 | 2 | 271 (144,1325) |
|  |  | 2011 | 4 | 4 | 344 (207,882) |
|  |  | 2012 | 8 | 6 | 241 (162,433) |
| Update Allele Frequencies  Inbreeding  Polygamy Males/Females  Scale Full Sib-ship  Sib-ship Prior  Unknown/Known population allele freq | 0  0  0 1  1  0  1 |  |  |  |  |
|  |  | 2010 | 2 | 2 | 271 (145,1452) |
|  |  | 2011 | 4 | 4 | 344 (204,920) |
|  |  | 2012 | 8 | 6 | 241 (162,433) |
| Update Allele Frequencies  Inbreeding  Polygamy Males/Females  Scale Full Sib-ship  Sib-ship Prior  Unknown/Known population allele freq | 0  1  0 1  1  0  1 |  |  |  |  |
|  |  | 2010 | 2 | 2 | 271(145,1452) |
|  |  | 2011 | 4 | 4 | 344 (205,903) |
|  |  | 2012 | 8 | 6 | 241 (166, 408) |
| Update Allele Frequencies  Inbreeding  Polygamy Males/Females  Scale Full Sib-ship  Sib-ship Prior  Unknown/Known population allele freq | 0  0  0 0  1  0  1 |  |  |  |  |
|  |  | 2011 | 4 | 4 | 344 (205,903) |
| Update Allele Frequencies  Inbreeding  Polygamy Males/Females  Scale Full Sib-ship  Sib-ship Prior  Unknown/Known population allele freq | 0  1  0 1  1  0  0 |  |  |  |  |
|  |  | 2011 | 4 | 2 | 344 (203,997) |
|  |  | 2012 | 8 | 5 | 241(157,415) |
| Update Allele Frequencies  Inbreeding  Polygamy Males/Females  Scale Full Sib-ship  Sib-ship Prior  Unknown/Known population allele freq  Precision 0/1/2/3 low/med/high/very-high | 0  1  0 1  1  0  0  2 |  |  |  |  |
|  |  | 2011 | 4 | 2 | 344 (203,997) |

# Supplementary Appendix S5

**Figure S5.1.** Boxplot of the estimated total length (TL) of samples determined using Equation 1 and the year-of-birth cohort (sampling effort per cohort), where each series of plots in faceted by year-of-capture. Year of birth cohort (x-axis) was calculated using age (determined using VBGF in Equation 2) minus the year-of-capture.

# Supplementary Appendix S6

**Table S6.1** Genetic diversity of EAP *C. carcharias* at 19 microsatellite loci: N (number of successfully genotyped individuals per locus); Na (number of alleles at each locus); Ho (observed heterozygosity), He (expected heterozygosity) and Fst (calculated as *Dst/Ht*, see Goudet, Jombart, & Goudet, 2015).

| **Locus** | **N** | **HWE Pr(ChiSq)** | **Ho** | **He** | **Fst** | |
| --- | --- | --- | --- | --- | --- | --- |
| Cca1419 | 185 | 0.000000 | 0.342 | 0.338 | | 0.007 |
| Cca83 | 185 | 0.243751 | 0.746 | 0.7 | | 0.011 |
| Cca1536 | 185 | 0.000000 | 0.756 | 0.721 | | -0.003 |
| Cca 1273 | 185 | 0.901786 | 0.842 | 0.827 | | -0.002 |
| Cca 1 | 185 | 0.017696 | 0.534 | 0.519 | | -0.005 |
| Cca 711 | 185 | 0.956946 | 0.378 | 0.306 | | 0.006 |
| Cca 1072 | 185 | 0.002555 | 0.834 | 0.813 | | 0 |
| Cca 627 | 185 | 0.000000 | 0.25 | 0.228 | | -0.003 |
| Cca 1466 | 185 | 0.001709 | 0.655 | 0.682 | | -0.001 |
| Cca 1276 | 185 | 0.518114 | 0.83 | 0.829 | | -0.001 |
| Cca 1226 | 185 | 0.965982 | 0.817 | 0.782 | | -0.007 |
| Iox10 | 184 | 0.872085 | 0.272 | 0.246 | | 0.035 |
| Ccar9 | 185 | 0.009576 | 0.631 | 0.466 | | -0.005 |
| Ccar13 | 185 | 0.572554 | 0 | 0 | | NA |
| CcaSA1 | 185 | 0.897367 | 0.401 | 0.351 | | 0.001 |
| CcaSA2 | 185 | 0.000034 | 0.507 | 0.513 | | 0.008 |
| CcaSA5 | 185 | 1.000000 | 0.342 | 0.338 | | 0.007 |
| CcaSA3 | 185 | 0.427863 | 0.746 | 0.7 | | 0.011 |
| CcaSA2 | 185 | 0.986457 | 0.756 | 0.721 | | -0.003 |
| Overall | 185 |  | 0.596 | 0.549 | |  |

# Supplementary Appendix S7

**Table S7.1** List of demographic, life-history and genetic priors used to initiate population-simulations for EAP of *C. carcharias*.

|  | **Prior name** | **Description** | | | **Prior values** | | | |  |  |  |  |  |  |
| --- | --- | --- | --- | --- | --- | --- | --- | --- | --- | --- | --- | --- | --- | --- |
| **Category: Demographic** | |  | | | |  | | |  |  |  |  |  |  |
|  | Population size (N) | An approximate total population size estimate (N), estimate of all individuals comprising the population | | | | 10,000 | | | | |  |  |  |  |
|  | Natural mortality rates | Estimates of the probability of individual mortality by age and sex, here we used the same values for male and female. | | | | 0.73 for YOY (Hillary et al., 2018), 0.786, 0.834, 0.825, 0.818, 0.812, 0.808, 0.804, 0.801, 0.799, 0.760, 0.676, 0.678, 0.679, 0.681, 0.683, 0.685, 0.687, 0.743, 0.747, 0.752, 0.756, 0.761, 0.765, 0.770, and then 0.809 until age 70. | | | | |  |  |  |  |
| **Category: Life-history** | |  | | | |  | | | |  |  |  |  |  |
|  | Maximum age | Longevity | | | | 73 | | | |  |  |  |  |  |
|  | Maximum mating age | The age of reproductive senescence | | | | 70 | | | | |  |  |  |  |
|  | Age of first reproduction |  | | | | 11 | | | | |  |  |  |  |
|  | Fecundity |  | | | | 10 | | | | |  |  |  |  |
| **Category: Genetic** | |  | |  | | |  | | | | |  | |  |
|  | Number of loci per individual | Number of genetic loci per individual available for interrogation; size of each individual’s simulated genome | | | | 100 | | | | |  |  |  |  |
| **Category:**  **Other Simulation parameters** | | | | | | |  |  | | | | |  |  |
|  | Simulation burn-in length | | Annual matings required to equilibrate demography and genetics | | | | 50 | | | |  |  |  |  |
|  | Simulation temporal evolution length | | Annual matings required for data gathering | | | | 50 | | | |  |  |  |  |
|  | Number of replicate simulations | | Number of populations independently generated with identical Scenario parameters | | | | 10 | | | |  |  |  |  |

Table S7.2 Summary of simulation results showing demographic $Nb, Ne$ lifetime mean ($\bar{k}$) and variance ($\bar{Vk}$) in reproductive success among individuals in a single cohort. Skip models are denoted with the number of cycles females were forced to forego reproduction. Here, 100 females were forced to skip each cycle, approximating 1/3rd of the total adult population size for applicable models.

| Model | Program | $Nb$ | $Ne$ | $\bar{Vk}$ | $\bar{k}$ | $Nb/Ne$ |
| --- | --- | --- | --- | --- | --- | --- |
| Standard | AgeNe | 372.7 | 857.2 | 60.63 | 2.00 | 0.43 |
| Standard | SimuPOP | 365.460 | 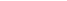  860.67 | 60.37 | 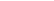  1.99 |   0.42 |
| 1-Skip | SimuPOP | 272.41 | 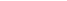  880.86  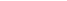 | 58.18 | 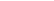  2.00  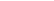 |   0.31   |
| 2-Skip | SimuPOP | 230.24 | 923.93 | 54.42 | 2.004 | 0.25 |

# References

Cailliet, G. M., Natanson, L. J., Welden, B. A., & Ebert, D. A. (1985). Preliminary studies on the age and growth of the white shark, Carcharodon carcharias, using vertebral bands. *Memoirs of the Southern California Academy of Sciences*, *9*(4).

Cailliet, G. M., Smith, W. D., Mollet, H. F., & Goldman, K. J. (2006). Age and growth studies of chondrichthyan fishes: the need for consistency in terminology, verification, validation, and growth function fitting. *Environmental Biology of Fishes*, *77*(3–4), 211–228. doi: 10.1007/s10641-006-9105-5

Domeier, M. (2012). A New Life-History Hypothesis for White Sharks, Carcharodon carcharias, in the Northeastern Pacific. In M. Domeier, *Global Perspectives on the Biology and Life History of the White Shark* (pp. 199–224). CRC Press. doi: 10.1201/b11532-19

Fitzpatrick, J. L., Kempster, R. M., Daly-Engel, T. S., Collin, S. P., & Evans, J. P. (2012). Assessing the potential for post-copulatory sexual selection in elasmobranchs. *Journal of Fish Biology*, *80*(5), 1141–1158. doi: 10.1111/j.1095-8649.2012.03256.x

Gubili, C. (2008). *Application of molecular genetics for conservation of the great white shark, Carcharodon carcharias, L. 1758*. University of Aberdeen.

Hillary, R. M., Bravington, M. V., Patterson, T. A., Grewe, P., Bradford, R., Feutry, P., … Bruce, B. D. (2018). Genetic relatedness reveals total population size of white sharks in eastern Australia and New Zealand. *Scientific Reports*, *8*(1). doi: 10.1038/s41598-018-20593-w

Holmes, B. J., Pope, L. C., Williams, S. M., Tibbetts, I. R., Bennett, M. B., & Ovenden, J. R. (2018). Lack of multiple paternity in the oceanodromous tiger shark ( *Galeocerdo cuvier* ). *Royal Society Open Science*, *5*(1), 171385. doi: 10.1098/rsos.171385

Jombart, T., Devillard, S., & Balloux, F. (2010). Discriminant analysis of principal components: a new method for the analysis of genetically structured populations. *BMC Genetics*, *11*(1), 94. doi: 10.1186/1471-2156-11-94

Li, H. (2014). Toward better understanding of artifacts in variant calling from high-coverage samples. *Bioinformatics*, *30*(20), 2843–2851. doi: 10.1093/bioinformatics/btu356

Luu, K., Bazin, E., & Blum, M. G. B. (2017). pcadapt : an R package to perform genome scans for selection based on principal component analysis. *Molecular Ecology Resources*, *17*(1), 67–77. doi: 10.1111/1755-0998.12592

O’Connor, J. (2011). *Age, Growth and Movement Signatures of the White Shark(Carcharodon Carcharias) in Southern Australia. School of the Environment, T*. The University of Technology, Sydney.

Smart, J. J., Chin, A., Tobin, A. J., & Simpfendorfer, C. A. (2016). Multimodel approaches in shark and ray growth studies: strengths, weaknesses and the future. *Fish and Fisheries*, *17*(4), 955–971. doi: 10.1111/faf.12154

Tanaka, S., Kitamura, T., Mochizuki, T., & Kofuji, K. (2011). Age, growth and genetic status of the white shark (Carcharodon carcharias) from Kashima-nada, Japan. *Marine and Freshwater Research*, *62*(6), 548. doi: 10.1071/MF10130

Von Bertalanffy, L. (1938). A quantitative theory of organic growth (inquiries on growth laws. II). *Human Biology*, *10*(2), 181–213.

Wintner, S. P. (1999). Age and growth determination of the white shark, Carcharodon carcharias, from the east coast of South Africa. *Fish. Bull*, *97*, 153–169.
